# Social media addiction as a mediator of the associations between fear of COVID-19, mental health symptoms, and problematic alcohol use

**DOI:** 10.3389/fpsyt.2023.1268890

**Published:** 2023-11-14

**Authors:** Jeremy W. Luk, Daniel W. Geda, Bethany L. Stangl, Cecilia Cheng, Melanie L. Schwandt, David Goldman, Nancy Diazgranados, Vijay A. Ramchandani

**Affiliations:** ^1^National Institute on Alcohol Abuse and Alcoholism, Bethesda, MD, United States; ^2^Human Psychopharmacology Laboratory, National Institute on Alcohol Abuse and Alcoholism, Bethesda, MD, United States; ^3^Social and Health Psychology Laboratory, Department of Psychology, The University of Hong Kong, Hong Kong, Hong Kong SAR, China; ^4^Laboratory of Neurogenetics, National Institute on Alcohol Abuse and Alcoholism, Rockville, MD, United States

**Keywords:** alcohol, behavioral addiction, stress and coping, fear of COVID-19, mental health, social media

## Abstract

**Background:**

Fear of COVID-19 is a risk factor for anxiety and depressive symptoms. During the COVID-19 pandemic, drinking to cope with psychological distress has been proposed as a key mechanism leading to problematic drinking. The goal of this study was to test social media addiction as a mediator linking fear of COVID-19 to mental health symptoms and problematic alcohol use.

**Methods:**

In between April 6 and July 2 of 2022, 250 participants completed an online survey as part of the National Institute on Alcohol Abuse and Alcoholism COVID-19 Pandemic Impact on Alcohol Study. Path analyses were conducted to test the mediational pathways.

**Results:**

Using the polythetic classification scheme, 13.2% (*n* = 33) of participants were classified as having social media addiction. Compared with participants without social media addiction, participants with social media addiction spent significantly more time on social media platforms and on digital communications with a family member or friend. They also reported greater fear of COVID-19, higher anxiety symptoms, and higher depressive symptoms. Path analyses indicated that social media addiction mediated the associations of fear of COVID-19 with anxiety and depressive symptoms. Furthermore, there were indirect pathways linking fear of COVID-19 to problematic alcohol use through higher social media addiction and higher anxiety and depressive symptoms.

**Conclusion:**

Social media addiction may be a maladaptive coping mechanism that individuals with high fear of COVID-19 utilized to deal with uncertainty and perceived risks during the pandemic. Findings underscore the need to examine cognitions related to fear of COVID-19 and address excessive social media use in the context of mental health and alcohol interventions.

## 1. Introduction

The COVID-19 pandemic has brought about significant changes to the way people live, work, and interact with others (1, 2). Social isolation attributable to the COVID-19 pandemic has contributed to lifestyle changes, such as reduced physical activities, disruptions of sleep, and a decline in social and leisure activities (3). As mitigation strategies against COVID-19 infections, digital learning and remote work have been utilized in educational and occupational settings to help individuals stay connected in the absence of physical contact (4, 5). Virtual interactions such as voice call, group call, and online messaging are also common during the COVID-19 pandemic and are associated with slight increase in feelings of positive affect and social connection (6). Nevertheless, relative to social support on social media, real life social support was a stronger and more consistent protective factor against a sense of social isolation, anxiety symptoms, and depressive symptoms (7). Moreover, while social media use can help connect people from different places and promote psychological health and wellbeing (8–10), excessive social media use can be problematic and is associated with mental health issues such as loneliness, low self-esteem, and depression (11).

### 1.1. Prevalence of social media addiction and public health relevance of associated outcomes

Social media addiction has been conceptualized as a type of behavioral addiction sharing symptoms that commonly underlie addictive behaviors, including salience, tolerance, mood modification, relapse, withdrawal, and interference with daily functioning (12, 13). In a global meta-analysis of studies that utilized the Bergen Social Media Addiction Scale (BSMAS) prior to the pandemic, Cheng and colleagues showed that prevalence estimates of social media addiction ranged from around 13–25% when using the moderate/severe cutoff or various polythetic classification schemes (14). Pandemic-related factors including lockdown and social isolation, financial hardship, mental health symptoms, and co-occurring problematic substance use have been identified as possible reasons that put individuals at risk for Internet-based behavioral addictions during the COVID-19 pandemic (15). In one meta-analysis of the COVID-19 literature, Casale and colleagues found that except in low-income countries, the prevalence of social media addiction did not increase significantly during the pandemic (16). In another meta-analysis, Alimoradi and colleagues estimated the prevalence of social media addiction during the COVID-19 pandemic to be 15.1% (17). Taken together, while multiple risk factors for social media addiction were present during the COVID-19 pandemic, the prevalence of social media addiction may have remained stable based on recent meta-analyses. Yet, more research is needed to document the prevalence of social media addiction across different samples using multiple commonly utilized classification methods (14), especially during the later phases of the pandemic.

According to the World Health Organization, the global prevalence of anxiety and depression had increased by 25% during the first year of the COVID-19 pandemic (18). Based on data from the National Center for Health Statistics, the number of alcohol-related deaths had also increased by 25% from 2019 to 2020 (19). These findings highlight the public health significance of understanding risk pathways leading to anxiety, depression, and problematic drinking in the context of the COVID-19 pandemic. Problematic social media use is known to increase risk for depressive symptoms (20), and social media addiction specifically has been associated with worse mental health symptoms during the pandemic (21–23). However, few studies have expanded the scope of investigation to understand potential precursors and consequences of social media addiction and mental health symptoms, such as fear of COVID-19 as a distal risk indicator and problematic alcohol use as a maladaptive coping strategy to deal with psychological distress experienced during the pandemic.

### 1.2. Conceptualization of mediation model linking fear of COVID-19 to clinical outcomes

Multiple forms of COVID-related anxiety have been documented in the literature, including anxiety over getting infected personally, concerns over others’ health and reactions, and worries about the pandemic’s negative societal and financial impact (24). Aspects of the fear response to the COVID-19 pandemic, such as danger and contamination fear, xenophobia stress, and compulsive checking stress, can also be considered as part of a broader COVID-related stress construct (25, 26). Fear of COVID-19 has been studied extensively as a contributor to adverse mental health outcomes during the pandemic (27, 28). According to a scoping review of this literature, 18.1–45.2% of individuals in various cohorts collected during the COVID-19 pandemic endorsed fear of COVID-19, and this fear was more prevalent among those in suspicion of being infected and those with mental health problems (29). Research conducted during the early phase of the pandemic showed that relatedness need frustration was a motivator for individuals to engage in social media addiction, which in turn led to higher levels of depressive symptoms and loneliness (30).

Social media addiction could be exacerbated during the pandemic as individuals may have reduced in-person gatherings out of fear of COVID-19 and may have engaged in problematic social media use to fulfill their need for social connection (15, 30). Consistent with this conceptualization, Brailovskaia and Margraf (31) found that higher COVID-19 burden related to everyday life restriction and constrained social situation was associated with a lower sense of control, which in turn was associated with increased social media addiction during the pandemic. Fear of COVID-19 may similarly trigger a reduced sense of control which can lead to the use of social media addiction as a maladaptive coping mechanism during the pandemic (32, 33). Accordingly, social media addiction may be an important mediator linking fear of COVID-19 to mental health symptoms. In a serial mediation analysis, Kayis et al. (34) found that fear of COVID-19 had both a direct effect on mental wellbeing and an indirect effect on mental wellbeing via loneliness and smartphone addiction. However, there is a lack of parallel research that tests social media addiction as a mediator of the negative impact of COVID-19 fear on mental health symptoms. Building on prior studies reviewed above, we theorized social media addiction as a maladaptive coping pathway that links fear of COVID-19 to anxiety symptoms and depressive symptoms.

Alcohol misuse is a significant public health issue during the COVID-19 pandemic (35), especially among individuals with a history of alcohol use disorder (AUD) (36–38). It is projected that increases in alcohol consumption attributable to the COVID-19 pandemic would lead to 295,000 more alcohol-related hospitalization and $5.4 billion over a 5-year period (39). A recent meta-analysis showed substantial heterogeneity in changes in alcohol use from before to during the pandemic (40). Change in media use has been positively associated with change in substance use during the COVID-19 pandemic (41). Moreover, use of alcohol to cope with psychological distress and COVID-related stressors has been proposed and demonstrated in multiple studies (42–46). Extending these studies and building on the internalizing pathway model of alcohol use (47), problematic alcohol use could be a downstream outcome of heightened fear of COVID-19, social media addiction, and mental health symptoms.

### 1.3. Scope of the current study

In this study, we examined the prevalence and correlates of social media addiction at a later phase of the COVID-19 pandemic after the peak of the Omicron variant wave (April 2022 to June 2022). Specifically, we tested social media addiction as a mediational pathway linking fear of COVID-19 to mental health symptoms and problematic alcohol use. We hypothesized that social media addiction would be associated with more time spent on social media platforms and digital communications, but less time spent on in-person interactions with friends or family. We also hypothesized that social media addiction would be positively associated with fear of COVID-19, mental health symptoms, and problematic alcohol use. Finally, we hypothesized that social media addiction would mediate the links between fear of COVID-19 and the mental health and alcohol-related outcomes.

## 2. Methods

### 2.1. Participants

A total of 265 participants completed an online survey in between April 6 and June 2 of 2022 as a follow-up to the National Institute on Alcohol Abuse and Alcoholism COVID-19 Pandemic Impact on Alcohol Study (NIAAA C19-PIA Study). Recruitment strategy and details of the larger study cohort can be found in our prior publications (38, 46, 48). Of the 265 participants who participated in this follow-up survey around the 2-year anniversary of the pandemic, 15 participants were excluded due to missing data on history of alcohol use disorder (*n* = 4) or clinical variables of interest (*n* = 11), yielding an analytic sample of 250 participants. All study participants provided informed consent. The C19-PIA Study protocol was approved by the National Institutes of Health Intramural Institutional Review Board and is registered in clinicaltrials.gov (NCT04391816).

### 2.2. Measures

Study variables are presented in the following order. First, social engagement variables are described as these items illustrate participants’ use of different methods to stay socially engaged with family and friends during the COVID-19 pandemic. Second, the predictor, mediator, and the three clinical outcomes in the mediation model were described. Third, measures that were essential to describe the study sample, such as History of AUD and COVID-19 Infection, were provided.

#### 2.2.1. Social engagement variables

Four items were taken from the Understanding America Study (49) and the response timeframe was adapted to reference the COVID-19 pandemic time period. Participants were asked to provide the best estimate of the number of days in a typical week during the COVID-19 pandemic that they were engaged in the following behaviors: (1) spent time posting or browsing on Facebook, Twitter, Instagram, or Snapchat; (2) messaged or emailed with a family member or friend; (3) had a phone call or video call with a family member or a friend; and (4) spent time interacting with a family member or friend in person.

#### 2.2.2. Fear of COVID-19

The Fear of COVID-19 Scale (FCV-19S) is a seven-item scale that measures fear responses in the context of the COVID-19 pandemic and has good reliability and validity properties (50). While the FCV-19S was initially validated in an Iranian sample, this scale has been translated into 16 different languages and used in 21 countries and its desirable psychometric properties have been reported repeatedly (51). Sample items include “I am most afraid of coronavirus-19,” “It makes me uncomfortable to think about coronavirus-19,” and “I am afraid of losing my life because of coronavirus-19.” Response options ranged from 0 “Strongly Disagree” to 2 “Neither Agree nor Disagree” and 4 “Strongly Agree.” A total score was calculated (Cronbach’s alpha = 0.88), with higher scores indicating increased fear of COVID-19.

#### 2.2.3. Social media addiction

The BSMAS is a six-item measure of social media addiction that has been widely used in the literature (12, 13). Its psychometric properties have been studied using representative samples from 9 countries and the BSMAS was found to be a reliable and valid instrument in the context of the COVID-19 pandemic (31). In this scale, participants were asked to rate how frequent they experienced symptoms of social media addiction in the past year. The 6 items include “Spent a lot of time thinking about social media or planned use of social media” (salience), “Felt an urge to use social media more and more” (tolerance), “Used social media to forget about personal problems” (mood modification), “Tried to cut down on the use of social media without success” (relapse), “Become restless or troubled if you have been prohibited from using social media” (withdrawal), and “Used social media so much that it has had a negative impact on your job/studies” (interference with daily functioning). Response options ranged from 1 “very rarely” to 3 “sometimes” and 5 “very often.” Three classification schemes, including the monothetic classification scheme (≥3 on all BSMAS items), a cut-off score of 18 on the BSMAS [as proposed in the initial validation study of the BSMAS (12, 52)], and the polythetic classification scheme (≥3 on at least 67% of the items), were utilized to identify participants with social media addiction (14).

#### 2.2.4. Anxiety symptoms

The Generalized Anxiety Disorder-7 Assessment (GAD-7) is a seven-item instrument assessing anxiety symptoms over the last 2 weeks (53). Sample items include “Feeling nervous, anxious or on edge,” “Trouble relaxing,” and “Becoming easily annoyed or irritable.” Response options ranged from 0 “Not at all” to 3 “Nearly every day.” A total score was calculated (Cronbach’s alpha = 0.92), with higher scores indicating higher anxiety symptoms.

#### 2.2.5. Depressive symptoms

The Patient Health Questionnaire-9 (PHQ-9) is a nine-item instrument assessing depressive symptoms over the last 2 weeks (54). Sample items include “Little interest or pleasure in doing things,” “Feeing down, depressed, or hopeless,” and “Feeing tired or having little energy.” Response options ranged from 0 “Not at all” to 3 “Nearly every day.” A total score was calculated (Cronbach’s alpha = 0.90), with higher scores indicating higher depressive symptoms.

#### 2.2.6. Problematic alcohol use

The Alcohol Use Disorders Identification Test (AUDIT) is a 10-item screening instrument developed by the World Health Organization to assess alcohol consumption, related harm, and dependence symptoms (55). We adapted the AUDIT to assess drinking in the last 3 months. Response options were scored on a scale from 0 to 4, yielding a maximum total of 40 (Cronbach’s alpha = 0.94).

#### 2.2.7. History of AUD and COVID-19 infection

AUD status was assessed using the Structural Clinical Interview for Diagnostic and Statistical Manual of Mental Disorders (56) and was obtained from the NIAAA natural history protocol database (57). History of COVID-19 infection was assessed using two questions: “Throughout the pandemic, how many times did you get tested for COVID-19?” and “Did you test positive for COVID-19?” Participants who were never tested for COVID-19 (*n* = 27, 10.8%) or did not test positive (*n* = 156, 62.4%) were coded as negative, and those who tested positive for COVID-19 were coded as positive (*n* = 67, 26.8%).

### 2.3. Statistical analysis

Data analyses followed four steps. First, we examined the prevalence of social media addiction using the monothetic classification scheme, a cut-off score of 18 on the BSMAS, and the polythetic classification scheme. Second, we examined demographic differences in social media addiction using a *t*-test and chi-square tests. Third, we examined social media addiction group differences in social engagement variables and clinical outcomes using linear regression models. Fourth, we conducted path analyses controlling for age, sex, and race to test social media addiction as a mediator of the associations between fear of COVID-19 and the clinical variables. We specified both the two-path mediation model (fear of COVID-19 → social media addiction → clinical outcomes) and the three-path mediation models (fear of COVID-19 → social media addiction → anxiety or depressive symptoms → problematic alcohol use). Bias-corrected 95% confidence interval bounds of the indirect effects were obtained using 10,000 bootstrap resamples to test significance of the mediational pathways (58).

To estimate the prevalence of social media addiction, three classification schemes were applied. The prevalence of social media addiction was 3.6% (*n* = 9) when using the monothetic classification scheme and 10.4% (*n* = 26) when using the cutoff score of ≥18 on the BSMAS. The prevalence of social media addiction was higher and estimated to be 13.2% (*n* = 33) when using the polythetic classification scheme. Based on a recent psychometric study, the polythetic classification scheme was preferred as it produced the optimal balance in terms of sensitivity, specificity, negative and positive predictive values when compared to the benchmark classification established using latent profile analysis (59). Thus, the polythetic classification was used to determine social media addiction status in subsequent analyses. Statistical analyses were conducted in Stata 17 and in Mplus 8.4.

## 3. Results

In this sample, 33 participants (13.2%) were classified as having social media addiction using the polythetic classification scheme. Table 1 shows sample characteristics for the overall sample and by social media addiction status. The study sample had a mean age of 47.3 years (SD = 14.6), was evenly split between females and males, and was diverse in terms of race/ethnicity and household income. About a quarter of the participants (26.8%) reported a history of COVID-19 infection and close to one-third of the participants (32.0%) reported a history of AUD. Participants with social media addiction were younger (mean age = 39.7 vs. 48.4 years, *p* = 0.001) and more likely to be female (66.7% vs. 47.5%, *p* = 0.04) than participants without social media addiction. Other demographic characteristics, household income, history of COVID-19 infection, and history of AUD were not associated with social media addiction.

**Table 1:** Sample characteristics for the overall sample and by social media addiction status.

	Overall (*N* = 250)		No social media addiction (*n* = 217; 86.8%)		With social media addiction (*n* = 33; 13.2%)			
	Mean	SD	Mean	SD	Mean	SD	*t*	*p*
Age	47.3	14.6	48.4	14.3	39.7	14.2	−3.272	0.001
	Frequency	Percent	Frequency	Percent	Frequency	Percent	*χ^2^*	*p*
Sex								
Female Male	125 125	50.0 50.0	103 114	47.5 52.5	22 11	66.7 33.3	4.224	0.040
Race								
White Black/African Other	132 79 39	52.8 31.6 15.6	115 70 32	53.0 32.3 14.8	17 9 7	51.5 27.3 21.2	1.005	0.605
Ethnicity								
Not Hispanic Hispanic Unknown	221 20 9	88.4 8.0 3.6	192 16 9	88.5 7.4 4.2	29 4 0	87.9 12.1 0	2.177	0.337
Household income								
< $20,000 $20,000–$74,999 $75,000–$149,999 $150,000 or more	59 76 67 48	23.6 30.4 26.8 19.2	52 64 57 44	24.0 29.5 26.3 20.3	7 12 10 4	21.2 36.4 30.3 12.1	1.703	0.636
History of COVID-19 infection								
No Yes	183 67	73.2 26.8	159 58	73.3 26.7	24 9	72.7 27.3	0.004	0.948
History of alcohol use disorder								
No Yes	170 80	68.0 32.0	149 68	68.7 31.3	21 12	63.6 36.4	0.333	0.564

**Figure 1:**
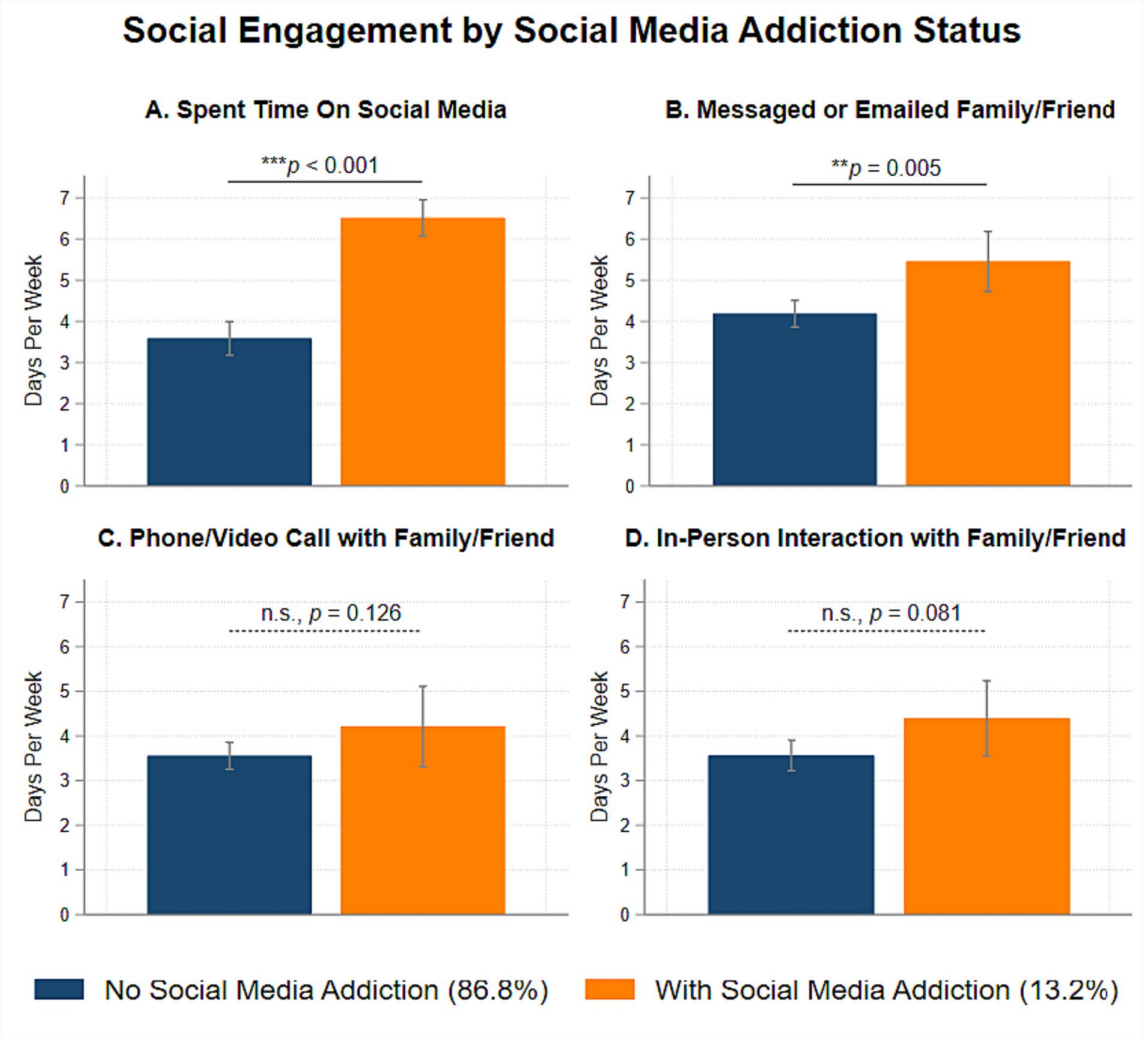
Associations between polythetic scheme classified social media addiction and social engagement variables during the COVID-19 pandemic.

Figure 1 shows the associations between social media addiction and social engagement variables. Relative to participants without social media addiction, participants with social media addiction spent more days in a typical week posting and browsing on social media platforms (*b* = 2.93, *SE* = 0.54, *p* < 0.001) and messaging or emailing with a family member or friend (*b* = 1.27, *SE* = 0.45, *p* = 0.005). Participants with and without social media addiction did not differ significantly in terms of days in a typical week spent on phone or video call with a family member or friend (*b* = 0.66, *SE* = 0.43, *p* = 0.126) and time spent on in-person interaction with a family or a friend (*b* = 0.83, *SE* = 0.47, *p* = 0.081).

**Figure 2:**
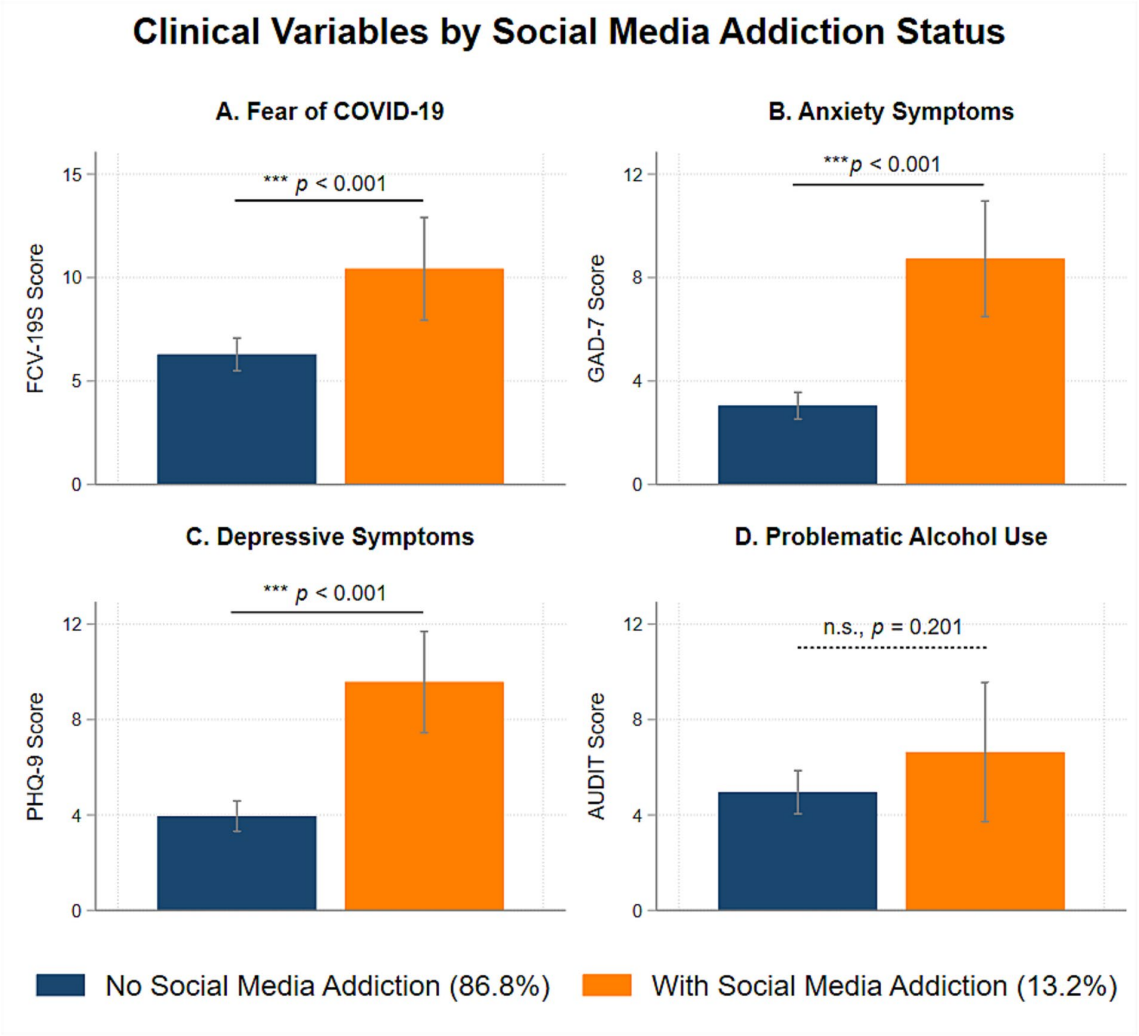
Associations between polythetic scheme classified social media addiction and clinical variables during the COVID-19 pandemic.

Figure 2 shows the associations between social media addiction and clinical variables. Relative to participants without social media addiction, participants with social media addiction reported greater fear of COVID-19 (*b* = 4.14, *SE* = 1.14, *p* < 0.001), higher anxiety symptoms (*b* = 5.68, *SE* = 0.80, *p* < 0.001), and higher depressive symptoms (*b* = 5.63, *SE* = 0.93, *p* < 0.001). Considering the direct association, participants with and without social media addiction did not differ significantly in terms of problematic alcohol use (*b* = 1.68, *SE* = 1.31, *p* = 0.201).

**Figure 3:**
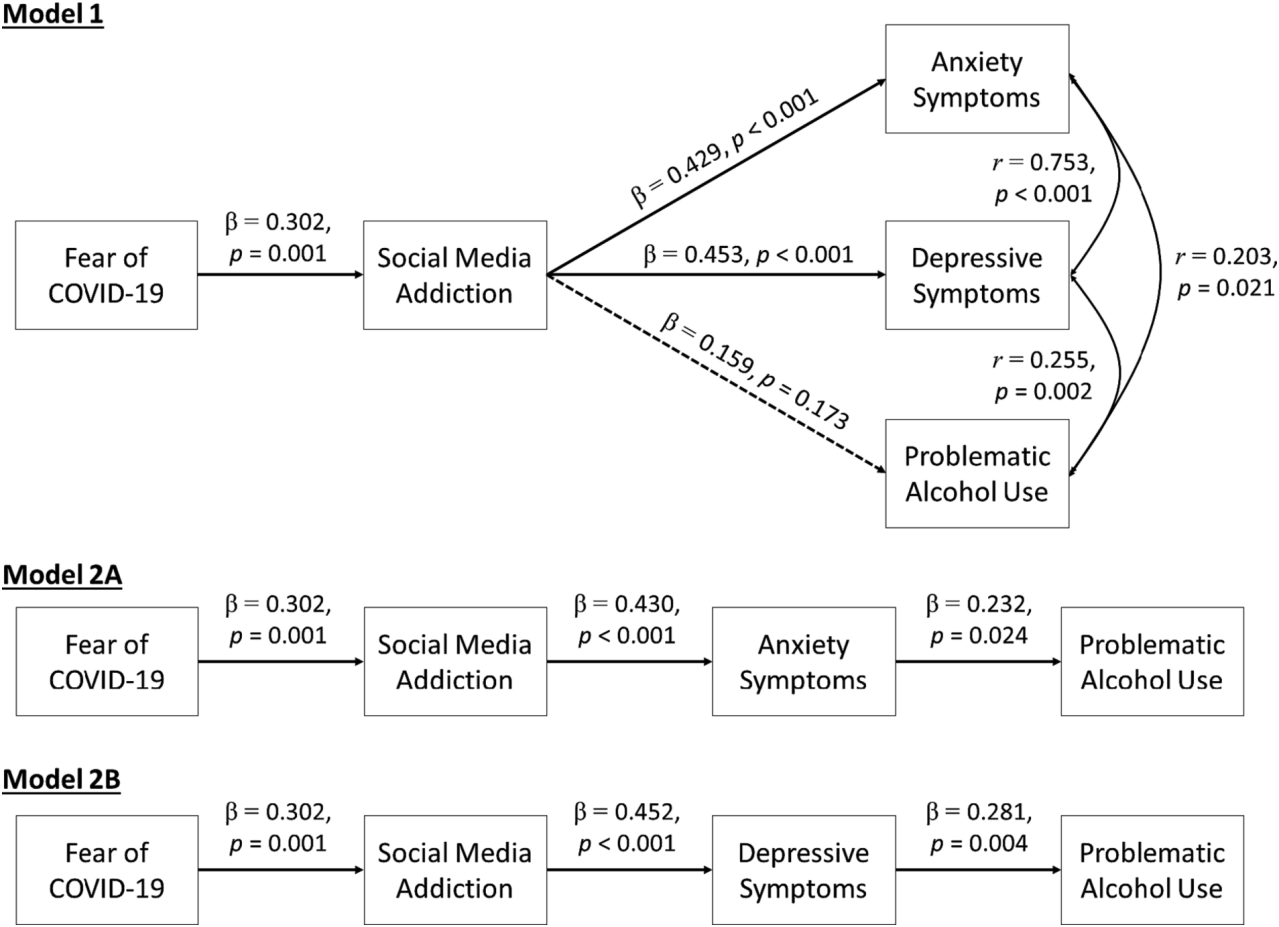
Social media addiction as a mediator linking fear of COVID-19 to mental health and problematic alcohol use.

Findings from mediation analyses are presented in Figure 3. The two-path mediation model (Model 1) shows that fear of COVID-19 was positively associated with social media addiction (*β* = 0.30, *SE* = 0.09, *p* = 0.001), which in turn was associated with higher anxiety symptoms (*β* = 0.43, *SE* = 0.09, *p* < 0.001) and depressive symptoms (*β* = 0.45, *SE* = 0.09, *p* < 0.001), but not problematic alcohol use (*β* = 0.16, *SE* = 0.12, *p* = 0.173). Due to the moderately high correlations between anxiety and depressive symptoms (*r* = 0.75), the three-path mediation models were estimated separately for anxiety symptoms (Model 2A) and depressive symptoms (Model 2B). These analyses indicated that participants with higher fear of COVID-19 were more likely to have social media addiction, which in turn was associated with higher problematic alcohol use via the internalizing mechanisms of anxiety or depressive symptoms. A summary of the indirect, direct, and overall total effects for the mediation models is presented in Table 2.

**Table 2:** Summary of indirect, direct, and overall total effects of fear of COVID-19 on mental health and problematic alcohol use.

Mediational pathways	Effects	*β* (bias-corrected 95% confidence intervals)
Model 1		
Fear of COVID-19 → SMA → anxiety symptoms	Indirect	**0.130 (0.048, 0.229)**
	Direct	**0.182 (0.053, 0.306)**
	Overall total	**0.312 (0.180, 0.440)**
Fear of COVID-19 → SMA → depressive symptoms	Indirect	**0.137 (0.049, 0.263)**
	Direct	0.081 (−0.067, 0.226)
	Overall total	**0.218 (0.092, 0.358)**
Fear of COVID-19 → SMA → problematic alcohol use	Indirect	0.048 (−0.022, 0.132)
	Direct	0.021 (−0.103, 0.151)
	Overall total	0.069 (−0.047, 0.191)
Model 2A		
Fear of COVID-19 → SMA → anxiety symptoms → problematic alcohol use	SMA indirect	0.018 (−0.071, 0.114)
	Anxiety indirect	**0.042 (0.006, 0.109)**
	SMA + anxiety indirect	**0.030 (0.006, 0.083)**
	Total indirect	**0.091 (0.023, 0.185)**
	Direct	−0.022 (−0.142, 0.105)
	Overall total	0.069 (−0.047, 0.193)
Model 2B		
Fear of COVID-19 → SMA → Depressive Symptoms → Problematic Alcohol Use	SMA Indirect	0.010 (−0.082, 0.099)
	Depression Indirect	0.023 (−0.015, 0.083)
	SMA + depression indirect	**0.038 (0.010, 0.100)**
	Total indirect	0.071 (−0.004, 0.171)
	Direct	−0.002 (−0.126, 0.127)
	Overall total	0.069 (−0.047, 0.193)

In mediation analyses, polythetic scheme classified social media addiction (SMA) was treated as a binary variable and the models were estimated using the Weighted Least Square Mean and Variance Adjusted (WLSMV) estimator. Standardized estimates of the indirect, direct, and overall total effects are presented. Significant effects are highlighted in bold.

## 4. Discussion

In this study, 13.2% of participants had social media addiction as classified by the polythetic scheme and social media addiction was more common among females and younger participants. This prevalence estimate is comparable to the 15.1% reported during the COVID-19 pandemic according to a recent meta-analysis (17). In a comprehensive global meta-analysis of pre-pandemic social media addiction, the prevalence of social media addiction as classified by the polythetic scheme was estimated to be 24% and the prevalence of social media addiction in individualistic cultures was estimated to be 14% compared to 31% in collectivistic cultures (14). Thus, the relatively low prevalence of social media addiction in this study may reflect a better age representation than prior studies and the sampling from an individualistic cultural context.

As expected, participants with social media addiction reported more time spent on social media platforms and on messaging and emailing family or friends than participants without social media addiction. These results suggest a preference among individuals with social media addiction to communicate with others using electronic means such as messaging or emailing. This finding makes intuitive sense as several major social media platforms also enable instant messaging. However, contrary to our expectations, there were no social media addiction group differences in phone/video call and in-person interactions with family or friends. One possible explanation is that individuals with social media addiction may be more extraverted (60, 61). Thus, engagement in social media addiction does not necessarily reduce their time spent on calling or interacting with their family or friends. As a future direction, it would be important to not only assess for the number of days spent on various social engagement behaviors, but also the length and quality of each of these social encounters.

Recent research has identified COVID-related anxiety as a contributor to social media addiction (62, 63). We replicated and extended this finding by illustrating social media addiction as a mediator of fear of COVID-19 and mental health outcomes. One interpretation is that individuals with higher fear of COVID-19 may be more inclined to remain home or stay isolated, reduce prosocial pleasant activities, and engage in excessive social media use, leading to higher anxiety and depressive symptoms. This interpretation is consistent with the conceptualization of problematic social media use as a maladaptive coping strategy during the pandemic (32). Clinically, behavioral health professionals can help individuals develop adaptive coping strategies (64) and minimize the negative impact of overabundant information and misinformation disseminated through social media on mental health related outcomes during the COVID-19 pandemic (65, 66).

Few studies tested the links between fear of COVID-19 and problematic alcohol use during the pandemic. In a recent study, Lac (67) utilized a COVID-19 stress measure and found that danger and contamination stress was inversely associated with drinking quantity and alcohol craving, whereas xenophobia stress and traumatic symptoms stress were positively associated with these alcohol-related outcomes. The lack of an overall association between fear of COVID-19 and problematic alcohol use in our study reflects the existence of multiple pathways that operate in opposite directions. Indeed, when social media addiction and internalizing symptoms were evaluated in the three-path mediation models, we identified an internalizing pathway through which fear of COVID-19 conferred risk on problematic alcohol use indirectly via social media addiction and anxiety/depressive symptoms. Consistent with prior research documenting drinking to cope during the pandemic (42–44, 46), individuals who engage in problematic social media use and exhibit negative mood symptoms out of fear of COVID-19 may be vulnerable to using alcohol to cope with COVID-related stress.

Despite its novelty, this study has several limitations. First, this study utilized a convenience sample and so generalization of the findings are limited. Replication with larger and representative samples is recommended. Second, measures of fear of COVID-19 and social media addiction were not initially collected in the baseline survey of the C19-PIA study. Thus, longitudinal analyses with the NIAAA C19-PIA Study cannot be conducted to estimate the mediation models. Third, due to the small sample size, social media addiction utilizing the monothetic and 18 or more cut off yielded low prevalence and analyses with these alternative classification schemes were not conducted due to limited statistical power. Fourth, the cross-sectional data limited inferences regarding direction of effects. While fear of COVID-19 could lead to social media addiction as implicated in a prior report of a serial mediation model (34), exposure to social media content and misinformation could also increase fear of COVID-19 (22). Longitudinal studies are needed to further disentangle the cause and effect in the associations in other datasets with repeated measures of these study variables.

## 5. Conclusion

During a later phase of the pandemic, social media addiction was reported among 13.2% of the study sample. Social media addiction was positively associated with time spent on social media platforms and digital communications with family or friends via messaging and emails. Mediation analyses revealed indirect associations linking fear of COVID-19 to mental health symptoms and problematic alcohol use via social media addiction. These findings highlight the relevance of addressing cognitions related to fear of COVID-19 and excessive social media use in the context of mental health and alcohol interventions during the COVID-19 pandemic and beyond. Future research is needed to examine how cognitive flexibility and adaptive coping can help individuals adjust to the “new normal” in the post pandemic world (68).

## Data availability statement

Data from this study are not publicly available due to ethical concerns regarding patient privacy and original patient consent. Data may be made available by requests directly to the corresponding authors.

## Ethics statement

The studies involving humans were approved by the NIH Intramural IRB (approval number: 20AA0115). The studies were conducted in accordance with the local legislation and institutional requirements. The participants provided their written informed consent to participate in this study.

## Author contributions

JL: Conceptualization, Formal analysis, Methodology, Visualization, Writing – original draft. DWG: Conceptualization, Writing – original draft. BS: Conceptualization, Writing - review & editing. CC: Conceptualization, Methodology, Writing – review & editing. MS: Conceptualization, Data curation, Writing – review & editing. DG: Conceptualization, Writing – review & editing. ND: Conceptualization, Funding acquisition, Project administration, Supervision, Writing – review & editing. VR: Conceptualization, Funding acquisition, Project administration, Supervision, Writing – review & editing.
